# Surfactant Protein C Deficiency in a Puerto Rican Adolescent With a Rare SFTPC Genetic Variant

**DOI:** 10.7759/cureus.17422

**Published:** 2021-08-24

**Authors:** Victor Acosta-Rivera, Jesus M Melendez-Montañez, Francisco Diaz-Sotomayor, Wilfredo De Jesús-Rojas

**Affiliations:** 1 Department of Internal Medicine, University of Massachusetts Medical School, Massachusetts, USA; 2 Department of Internal Medicine, Ponce Health Sciences University, Ponce, PRI; 3 School of Medicine, Ponce Health Sciences University, Ponce, PRI; 4 Department of Pediatrics, University of Puerto Rico, Medical Sciences Campus, San Juan, PRI; 5 Department of Pediatrics, Ponce Health Sciences University, Ponce, PRI

**Keywords:** surfactant protein c, sftpc mutations, childhood interstitial lung disease, rare lung disease, digital clubbing

## Abstract

Surfactant protein C (SP-C) is a hydrophobic lipoprotein necessary for lowering alveolar surface tension and lung defense mechanisms. Defects in its function due to genetic mutations in the *SFTPC* gene have been increasingly identified in patients presenting with childhood interstitial lung disease. *SFTPC* mutations are inherited in an autosomal dominant pattern with reduced penetration and variable expressivity, although *de novo* mutations have also been documented. In this article, we present the case of an oxygen-dependent 13-year-old male with interstitial lung disease and severe pulmonary hypertension. Genetic analysis and lung biopsy confirmed the diagnosis of SP-C deficiency with the rare heterozygous mutation IVS4+2. To our knowledge, this is the first documented case of SP-C deficiency in the Puerto Rican population and the second worldwide with the IVS4+2 genetic mutation.

## Introduction

Pulmonary surfactant is a lipoprotein exclusively produced and secreted by type II alveolar cells and is necessary for lowering alveolar surface tension and providing innate defense mechanisms against inhaled pathogens such as respiratory syncytial virus (RSV) [1,2]. To date, four distinct surfactant proteins (SP-A, SP-B, SP-C, and SP-D) have been identified [3]. The human *SFTPC* gene encodes for a large (21 kD) precursor SP-C (proSP-C) that requires processing into mature SP-C (3.7 kD) [4]. It has been proposed that mutations in the *SFTPC* gene lead to misfolding of the large proSP-C with subsequent accumulation in type II alveolar cells. This activates a pro-inflammatory cascade that triggers cellular death and recruitment of T-cells, along with fibroblasts, eventually leading to the development of childhood interstitial lung disease (chILD) [5]. These malfunctions or deficiencies in SP-C have been increasingly identified in previously unknown causes of chILD due to advancements in diagnostic technologies including genetic testing and histopathological analysis of lung tissue [6-12]. These mutations were first described by Nogee et al. [5] as having an autosomal dominant mode of inheritance with variable expressivity and penetration. However, *de novo* mutations are also commonly reported [10-12].

Clinical manifestations of SP-C deficiency are highly variable and may present with a range of nonspecific symptoms such as dyspnea, dry cough, exercise intolerance, and recurrent respiratory infections [13]. In more severe cases, children may develop acute respiratory distress triggered by respiratory viruses [13,14]. Symptoms usually spare the neonatal period and begin to manifest later in infancy [5]. As these patients grow older, other signs such as tachypnea, digital clubbing, hypoxia, resting or exercise-induced cyanosis, and failure to thrive may develop [11,13].

Clinical cases and evidence-based treatment options of SP-C deficiency due to *SFTPC* mutations are scant in the medical literature, especially in the Hispanic population. In this article, we report a case of chILD secondary to SP-C deficiency in a 13-year-old Puerto Rican male with a rare *SFTPC* genetic variant and discuss our treatment approach and multidisciplinary management strategies.

## Case presentation

**Figure 1:**
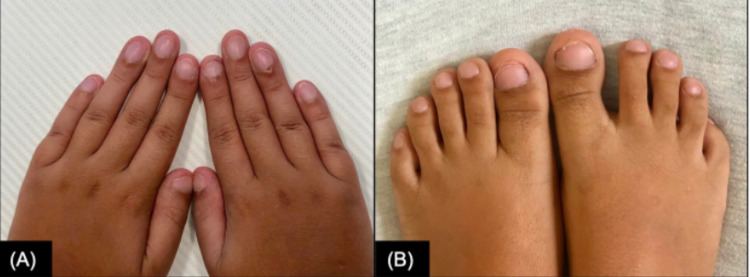
Generalized digital clubbing. Symmetric abnormal nail angulation of the hands (A) and toes (B) at 12 years of age.

A 13-year-old adolescent male presented with a history of progressive dyspnea on exertion, recurrent wheezing, bronchiolitis, pneumonia, and chronic respiratory failure since infancy. He was born at term (38 weeks, 6 days) via vaginal delivery and had no neonatal respiratory distress or complications. Family history was significant for asthma in his brother and one uncle. His mother had no significant past medical history and his father had expired six years prior due to a traumatic accident with no apparent medical history. Beginning at six months of age, the patient started to develop respiratory symptoms and had amassed 29 hospitalizations for respiratory distress, with eight of them requiring Pediatric Intensive Care Unit (PICU) admissions and two endotracheal intubations. Two weeks prior to our initial encounter, the patient was admitted to the PICU for four days due to an acute episode of shortness of breath and associated chest pain with hypoxemia. On initial presentation to our clinic, the patient required 24-hour oxygen supplementation via nasal cannula at 2 L per minute (LPM). Pulse oximetry was 92-95% with a nasal cannula on rest and decreased to 80-85% on room air with physical activity. Physical examination demonstrated a chronically ill-appearing 13-year-old boy with a heart rate of 114 beats per minute, respiratory rate of 25 breaths per minute, oxygen saturation of 92% at 2 LPM, body mass index of 18.16 (45th percentile), generalized digital clubbing of both upper and lower extremities (Figure 1), and lungs clear to auscultation bilaterally.

A complete pulmonary function test (PFT) was performed following the American Thoracic Society guidelines [15]. Forced vital capacity (FVC) was 58% predicted, forced expired volume in one second (FEV1) was 58% predicted, FEV1/FVC ratio was 100%, and DLCOcor was 32% predicted, consistent with a restrictive airflow pattern. A flexible fiberoptic bronchoscopy with bronchoalveolar lavage was performed which was remarkable for bronchial cells with reactive and inflammatory features as well as reactive macrophages, activated histiocytes, polymorphonuclear infiltrates with degenerative changes, fibrin, and amorphous material. The patient underwent a left lung wedge biopsy which showed bronchiectasis and cystic changes of airspace consistent with chILD. Periodic acid Schiff stain was negative. The immunology workup showed appropriate levels of immunoglobulins and normal CH50. Sweat test for cystic fibrosis and alpha-1-antitrypsin levels were within normal ranges. Cardiac echocardiography (ECHO) suggested evidence of pulmonary arterial hypertension (PAH) with right ventricle and pulmonary artery dilatation secondary to chronic lung disease. The maximal tricuspid regurgitation velocity on ECHO was 3.17 m/s (normal <2.5 m/s). Cardiac catheterization confirmed the diagnosis of severe PAH revealing baseline pulmonary arterial pressure of 64/34 mmHg with 10.2 WU m^2^ and a positive acute vasoreactivity testing with nitric oxide (NO) to 45/20 mmHg and 5.4 WU m^2^.

**Figure 2:**
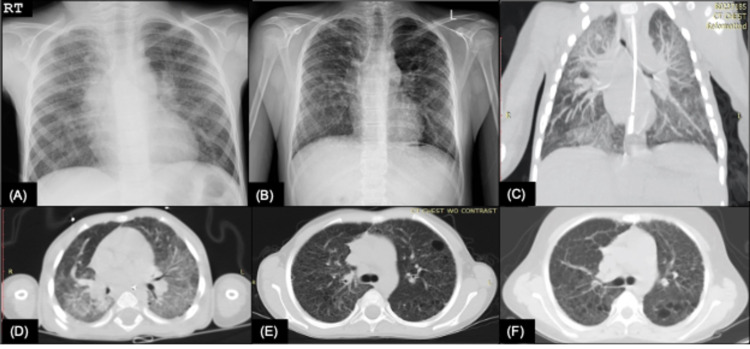
Progression of radiologic findings in a Puerto Rican male with SP-C deficiency. (A) AP view CXR taken at seven years of age showing central peribronchial wall thickening with bilateral diffuse interstitial changes. (B) PA view CXR taken at 13 years of age showing reticulonodular interstitial opacities. (C) Coronal and (D) transverse HRCT obtained at 14 months of age with evidence of significant ground-glass opacities, more prominent on the posterior lung aspects. (E) Transverse HRCT with decreased ground-glass opacity attenuation with a myriad of pulmonary cysts at six years of age and (F) 13 years of age. AP: anteroposterior; CXR: chest radiography; PA: posteroanterior; HRCT: high-resolution computed tomography

The earliest radiological imaging of the patient was a high-resolution computed tomography (HRCT) of the lungs performed at 14 months of age which was remarkable for evidence of marked, patchy ground-glass opacities (Figure 2, Panels C, D). When the patient was three years old, a follow-up HRCT demonstrated the development of a myriad of pulmonary cystic lesions. A subsequent scan performed when the patient was six years old continued to demonstrate diffuse pulmonary cysts of varying sizes, but with decreased attenuation of ground-glass opacities (Figure 2, Panel E). Simple chest X-rays showed bilateral and diffuse interstitial changes (Figure 2, Panels A, B).

The patient underwent genetic testing using a diagnostic genetic panel which was sequenced for 111 genes, including *ABCA3*, *CSF2RA*, *CSF2RB*, *FOXF1*, *NKX2-1*, *SFTPB*, and *SFTPC*. Genetic results were remarkable for one likely pathogenic variant in the *SFTPC* gene, IVS4+2, which is associated with autosomal dominant SP-C deficiency. All these findings were consistent with a diagnosis of *SFTPC*-related disorder.

Baseline dual-energy X-ray absorptiometry scan and ophthalmologic evaluation were obtained before treatment was initiated. The medication regimen included hydroxychloroquine 10 mg/kg/day at start dose for three months and 5 mg/kg/day as maintenance and azithromycin 500 mg three times a week as anti-inflammatory therapy. Verapamil 120 mg daily, sildenafil 20 mg three times a day, and bosentan 62.5 mg twice daily were added for pulmonary hypertension management. The patient was also started on serial intravenous pulses of methylprednisolone at 1 g/day for three doses on a monthly basis. Following three months of therapy, the patient tolerated breathing on room air and reserved his nasal cannula use for more demanding physical activities. Furthermore, the predicted DLCOcor increased to 38%. His most recent HRCT showed no progression of his pulmonary lung disease with persistent diffuse lung cysts (Figure 2, Panel F).

## Discussion

Surfactant proteins play a coordinating role in the synthesis, secretion, film formation, and recycling of phospholipids. SP-C has been postulated to modulate membrane-associated viral sensors such as toll-like receptor 3 [14]. Diseases that affect the production of these lipoproteins result in the development of chILD [13]. Previous studies have demonstrated that mutations that affect SP-B are incompatible with life if not corrected with bilateral lung transplantation [9,16]. However, given the normal pulmonary function of neonates and infants with *SFTPC* mutations, it is presumed that surfactant function remains stable until challenged by overwhelming inflammatory responses from common viral infections such as RSV [9,14]. This has been demonstrated with genetically modified SP-C null mice that exhibit decreased clearance of RSV with an associated prolonged inflammatory response. With the high prevalence and high reinfection rate of RSV, SP-C-deficient children suffer from increased and recurrent lung injury [14]. Furthermore, misfolded proSP-C accumulates in type II alveolar cells, leading to inflammation and cellular death. Destruction of type II alveolar cells prevents their normal function of replenishing type I alveolar cells after cellular injury. Ultimately, this leads to the development of pulmonary fibrosis, as seen in our case [1].

The phenotype of *SFTPC*-mutated patients is highly variable and depends little on the type of mutation, localization in the gene, or the age of onset. Even when different patients inherit the most prevalent *SFTPC* mutation, p.I73T (c.218T>C), the disease manifestation can vary from death in infancy to an asymptomatic carrier state [3,8]. This suggests that clinical presentation is likely multifactorial. As such, it is difficult to compare *SFTPC*­-mutated patients based on their genetic sequence alone [11].

To our knowledge, our case comprises the second known documented case of the IVS4+2 genetic variant. This mutation causes a sequence change (c.435+2T>C) on the *SFTPC* gene that induces an altered splice site that leads to a shortened protein product. IVS4+2 was discovered by van Moorsel et al. [10] in a 30-year-old Dutch patient with a personal history of ILD and a family history of pulmonary fibrosis. With reverse transcription-polymerase chain reaction (PCR) of the patient’s lung sample, they were able to demonstrate a truncated 200 bp product compared to the expected wild-type PCR product of 311 bp. Sequencing of the IVS4+2 PCR product revealed a lack of exon 4 of *SFTPC*. It cannot be established if the mutation in our patient was sporadic or part of a familial pulmonary fibrosis pattern because there is missing history and genetic sample from his father. The patient’s mother, however, tested negative for the presence of any *SFTPC* mutation.

Treatment of SP-C deficiency is derived from the overall management of chILD [16]. The therapeutic approach is mostly supportive therapy with empiric pharmacological treatment with corticosteroids, hydroxychloroquine, and azithromycin. Corticosteroids such as methylprednisolone pulse or oral prednisone are the mainstay treatments in chILD with inflammatory lung damage [13,16]. Hydroxychloroquine has been used successfully in several cases of SFTPC mutations. It is postulated to interfere and decrease the accumulation of aberrant proSP-C on type II alveolar cells [11,12,17]. In extreme cases, lung transplantation has been implemented, although this practice is more common in SP-B deficient patients [9,11,16]. No clinical studies on pharmacological treatment have been conducted specifically with SP-C deficient patients. Other strategies, such as surfactant replacement, only seem to provide transient relief and provide no long-lasting benefits [18]. Our patient also had evidence of severe PAH, which is not often detailed in case studies of SP-C deficiency. This was first seen on ECHO and later confirmed during cardiac catheterization. Verapamil, sildenafil, and bosentan were added to his medication regimen to control PAH as per the established guidelines [19].

Kroner et al. [11] reported that outcomes for treated *SFTPC*-mutated patients tend to be halting of disease progression, with significant improvement only seen in a minority of cases. Our patient continued this disease-halting trend given that complete PFTs following treatment managed to slightly increase DLCOcor parameters and there was no significant change on HRCT. This suggests the need for swift recognition of this disease to prevent irreversible lung damage. Given the high variability in clinical presentation, lack of standardization of treatment, wide side effect profile of medications, and multiple comorbidities associated with SP-C deficiency, a multidisciplinary approach that includes a variety of subspecialties should be established to manage clinically complex pediatric patients with rare pulmonary diseases (Table 1).

**Table 1:** Suggested multidisciplinary approach to manage SP-C deficiency in pediatrics. ECG: electrocardiogram; ACTH: adrenocorticotropic hormone; chILD: childhood interstitial lung disease; CBC: complete blood count; LFT: lung function test; DEXA: dual-energy X-ray absorptiometry; SARS-CoV-2: severe acute respiratory syndrome coronavirus 2

Subspeciality	Screening	Evaluation
Cardiology	Evaluate for the presence of congenital heart malformations or pulmonary arterial hypertension. Detection of prolonged QTc interval in view of long-term use of medications that affect QTc	2D echocardiography with tissue Doppler and color flow to evaluate heart function and detection of right heart strain. ECG prior to long-term use of hydroxychloroquine and azithromycin combination due to QTc prolongation and torsade de pointes. Cardiac catheterization for documentation of pulmonary arterial pressures, if clinically indicated
Endocrinology	Monitoring for the development of Cushing’s syndrome/adrenal suppression secondary to exogenous corticosteroid use and appropriate withdrawal from chronic steroid therapy. Provide recommendations about stress doses prior to surgeries or invasive procedures	Morning cortisol, serum ACTH, and glucose levels
Genetics	Diagnostic genetic testing for SFTPC gene mutations and genetic counseling about family planning. Rule out other genetic chILD disorders as part of the differential diagnosis	Referral to a genetic counselor for diagnostic discussion and prognostic and family planning
Nutrition	Monitor for height/weight to achieve a goal at the 50th percentile for age	Weight loss management strategies. Caloric intake adjustments to meet specific nutritional daily requirements
Ophthalmology	Screening for cataracts and glaucoma as a side effect of chronic steroid and hydroxychloroquine use	Ophthalmologic examination at baseline, followed by annually
Primary care	Detection of anemia, leukopenia, thrombocytopenia, or G6PD deficiency in view of chronic treatment with hydroxychloroquine. Screening for systemic hypertension	Baseline basic lab work (CBC, LFTs) prior to initiating hydroxychloroquine, baseline DEXA scan prior to corticosteroid therapy due to risk of osteopenia. Monitor blood pressure levels. Follow immunization schedule, including influenza, pneumococcal polysaccharide vaccine (PPSV23), and SARS-CoV-2 vaccine as per the American Academy of Pediatrics guidelines
Pulmonology	Screening for baseline and exertional hypoxemia, exercise intolerance, and evaluation airflow limitations. Documentation of abnormal pulmonary sounds on examination, and presence of clubbing. Assessment for lung transplantation, if needed	Baseline radiographic imaging of the chest X-ray or high-resolution computed tomography of the lungs. Record the percentage of oxygen saturation during every visit at rest and with activity. Serial spirometry every 3 months and complete pulmonary function test with DLCO every 6-12 months. Six-minute walk test to monitor for exertional hypoxemia
Sleep medicine	Screening of pediatric sleep disorders and nocturnal hypoxemia or hypoventilation	May consider diagnostic polysomnography with end-tidal CO_2_ and/or CPAP titration studies if evidence of obstructive sleep apnea is present. Management of sleep-related disorders

Ground-glass opacities either alone or mixed with small, diffuse lung cysts have been reported as the most frequent findings seen on HRCT of *SFTPC*-mutated patients [20]. Furthermore, it has been reported that, although nonspecific to SP-C deficiency, ground-glass opacities tend to decrease with age while lung cysts generally increase. Ground-glass opacities can be appreciated as early as nine months of age on all reported *SFTPC*-mutated cases [11,20]. Our patient presented with the same progression as evident by extensive ground-glass lesions seen on HRCT at 14 months of age (Figures 2C, 2D), and presented with a myriad of pulmonary cysts but scarcely appreciated ground-glass lesions on follow-up imaging at 6 and 13 years of age (Figures 2E, 2F). Mechri et al. [20] established a correlation between radiologic findings of HRCT with histopathology of lung biopsy. Ground-glass opacities on HRCT correlated with diffuse alveolar septal thickening, type II pneumocyte hyperplasia, and intra-alveolar accumulation of macrophages, while lung cysts correlated with dilation of the respiratory bronchiole and alveolar ducts. Our patient’s biopsy results were consistent with these findings.

*SFTPC* mutations and SP-C deficiency had not been previously documented in the Puerto Rican population. With better screening tools, previously unknown etiologies of ILD may become apparent. This grants the patient a more specific prognosis and disease-oriented management strategies [16]. Additionally, there is overall scarce data available for SP-C deficiency. Therefore, further identification and classification of *SFTPC *mutations are crucial to gain a better understanding of the disease, especially in pediatrics.

## Conclusions

We present the first known documented pediatric case of chILD due to SP-C deficiency in a Puerto Rican patient. Moreover, this is the second known case of the IVS4+2 genetic variant, and the first case described in a pediatric patient. Due to its variable phenotypic presentation, early screening with genetic testing for surfactant protein disorders, including *SFTPC* mutations, in patients presenting with ILD of unknown etiology should be considered.

## References

[1] Han S, Mallampalli RK (2015). The role of surfactant in lung disease and host defense against pulmonary infections. Ann Am Thorac Soc.

[2] Singh J, Jaffe A, Schultz A, Selvadurai H (2021). Surfactant protein disorders in childhood interstitial lung disease. Eur J Pediatr.

[3] Lyra PP, Diniz EM (2007). The importance of surfactant on the development of neonatal pulmonary diseases. Clinics (Sao Paulo).

[4] Mulugeta S, Nguyen V, Russo SJ, Muniswamy M, Beers MF (2005). A surfactant protein C precursor protein BRICHOS domain mutation causes endoplasmic reticulum stress, proteasome dysfunction, and caspase 3 activation. Am J Respir Cell Mol Biol.

[5] Nogee LM, Dunbar AE 3rd, Wert SE, Askin F, Hamvas A, Whitsett JA (2001). A mutation in the surfactant protein C gene associated with familial interstitial lung disease. N Engl J Med.

[6] Thomas AQ, Lane K, Phillips J 3rd (2002). Heterozygosity for a surfactant protein C gene mutation associated with usual interstitial pneumonitis and cellular nonspecific interstitial pneumonitis in one kindred. Am J Respir Crit Care Med.

[7] Brasch F, Griese M, Tredano M (2004). Interstitial lung disease in a baby with a de novo mutation in the SFTPC gene. Eur Respir J.

[8] Guillot L, Epaud R, Thouvenin G (2009). New surfactant protein C gene mutations associated with diffuse lung disease. J Med Genet.

[9] Hamvas A, Nogee LM, White FV (2004). Progressive lung disease and surfactant dysfunction with a deletion in surfactant protein C gene. Am J Respir Cell Mol Biol.

[10] van Moorsel CH, van Oosterhout MF, Barlo NP (2010). Surfactant protein C mutations are the basis of a significant portion of adult familial pulmonary fibrosis in a dutch cohort. Am J Respir Crit Care Med.

[11] Kröner C, Reu S, Teusch V (2015). Genotype alone does not predict the clinical course of SFTPC deficiency in paediatric patients. Eur Respir J.

[12] Kazzi B, Lederer D, Arteaga-Solis E, Saqi A, Chung WK (2018). Recurrent diffuse lung disease due to surfactant protein C deficiency. Respir Med Case Rep.

[13] Ferraro VA, Zanconato S, Zamunaro A, Carraro S (2020). Children's interstitial and diffuse lung diseases (ChILD) in 2020. Children (Basel).

[14] Glasser SW, Witt TL, Senft AP (2009). Surfactant protein C-deficient mice are susceptible to respiratory syncytial virus infection. Am J Physiol Lung Cell Mol Physiol.

[15] Graham BL, Steenbruggen I, Miller MR (2019). Standardization of Spirometry 2019 Update. An Official American Thoracic Society and European Respiratory Society Technical Statement. Am J Respir Crit Care Med.

[16] Kurland G, Deterding RR, Hagood JS (2013). An official American Thoracic Society clinical practice guideline: classification, evaluation, and management of childhood interstitial lung disease in infancy. Am J Respir Crit Care Med.

[17] Hepping N, Griese M, Lohse P, Garbe W, Lange L (2013). Successful treatment of neonatal respiratory failure caused by a novel surfactant protein C p.Cys121Gly mutation with hydroxychloroquine. J Perinatol.

[18] Hamvas A, Cole FS, deMello DE, Moxley M, Whitsett JA, Colten HR, Nogee LM (1994). Surfactant protein B deficiency: antenatal diagnosis and prospective treatment with surfactant replacement. J Pediatr.

[19] Rosenzweig EB, Abman SH, Adatia I (2019). Paediatric pulmonary arterial hypertension: updates on definition, classification, diagnostics and management. Eur Respir J.

[20] Mechri M, Epaud R, Emond S (2010). Surfactant protein C gene (SFTPC) mutation-associated lung disease: high-resolution computed tomography (HRCT) findings and its relation to histological analysis. Pediatr Pulmonol.

